# SPR-based fragment screening with neurotensin receptor 1 generates novel small molecule ligands

**DOI:** 10.1371/journal.pone.0175842

**Published:** 2017-05-16

**Authors:** Sylwia Huber, Fabio Casagrande, Melanie N. Hug, Lisha Wang, Philipp Heine, Lutz Kummer, Andreas Plückthun, Michael Hennig

**Affiliations:** 1 Roche Innovation Center Basel, Pharmaceutical Research and Early Development, Basel, Switzerland; 2 Department of Biochemistry, University of Zurich, Zurich, Switzerland; Cleveland Clinic Lerner Research Institute, UNITED STATES

## Abstract

The neurotensin receptor 1 represents an important drug target involved in various diseases of the central nervous system. So far, the full exploitation of potential therapeutic activities has been compromised by the lack of compounds with favorable physicochemical and pharmacokinetic properties which efficiently penetrate the blood-brain barrier. Recent progress in the generation of stabilized variants of solubilized neurotensin receptor 1 and its subsequent purification and successful structure determination presents a solid starting point to apply the approach of fragment-based screening to extend the chemical space of known neurotensin receptor 1 ligands. In this report, surface plasmon resonance was used as primary method to screen 6369 compounds. Thereby 44 hits were identified and confirmed in competition as well as dose-response experiments. Furthermore, 4 out of 8 selected hits were validated using nuclear magnetic resonance spectroscopy as orthogonal biophysical method. Computational analysis of the compound structures, taking the known crystal structure of the endogenous peptide agonist into consideration, gave insight into the potential fragment-binding location and interactions and inspires chemistry efforts for further exploration of the fragments.

## Introduction

Neurotensin receptor 1 (NTS1, also called NTR1, NTSR1) is a member of the β group of the class A GPCR family which is involved in dopaminergic, serotonergic and putative noradrenergic neurotransmission. NTS1 regulates many physiological (*e*.*g*. food uptake) and pathophysiological processes associated with Parkinson’s disease, schizophrenia or depression [1–4].

Crystal structures of *Rattus norvegicus* NTS1 receptor complexed with endogenous ligand, neurotensin (NT) peptide, were solved for thermostabilized receptor variants by two independent groups [4, 5]. White *et al*. [5] crystalized agonist-bound NTS1 receptor in complex with truncated neurotensin peptide NT_8-13_ and determined the structure at a resolution of 2.8 Å (PDB ID: 4GRV). The protein used here was a fusion to T4 lysozyme replacing intracellular loop 3, thermostabilized in the presence of agonist (NT) [5, 6] and requiring expression in insect cells, and lipidic cubic phase crystallization. Egloff *et al*. [4] crystalized different thermostabilized variants of NTS1 receptor obtained by directed molecular evolution in complex with truncated neurotensin peptide NT_8-13_ in a vapor diffusion approach (PDB IDs: 3ZEV, 4BUO, 4BV0, 4BWB). The highest resolution structure was determined at 2.75 Å, and the receptor was devoid of any bulky modification at the cytoplasmic face, thus preserving signaling activity. Here we concentrate on 4BWB, the variant most stable in the apo state. The structures 4GRV and 4BWB differ significantly in the ligand binding pocket, with 4BWB well supported by electron density, and by the presence of the amphipathic helix 8 in 4BWB, with its absence observed only in the T4L-fused structure 4GRV, as discussed previously [4].

G-protein coupled receptors with a neuropeptide binding site like the neurotensin receptor 1 represent a challenging class of drug targets [1]. Large efforts in pharmaceutical research have been invested to generate novel peptidic and non-peptidic compounds with promising preclinical data. So far, these data have not been successfully translated into clinical Phase II/III trials. For example, meclinertant (reminertant, SR48692), a selective non-peptidic NTS1 antagonist, failed to show convincing efficacy in schizophrenia [7–9]. Study and compound limitations, however, have precluded a definitive conclusion on the efficacy, and a full assessment of the receptor could not be achieved.[1] In particular, the brain penetration properties of the molecule have been questioned [1, 10].

Current treatment options of disorders mediated by NTS1 with the approved Pfizer compound PD149163, a derivative of the endogenous agonist neurotensin, show cognitive, antipsychotic and anxiolytic effects in preclinical as well as clinical experiments [2, 11]. PD149163 is reported as a selective and brain-penetrant NTS1 receptor agonist. To our knowledge, no data on brain penetration properties of PD149163 have been published, however; given the peptidic structure and the molecular weight of the compound, we anticipate that only a minor fraction of PD149163 reaches the receptors in the brain [12, 13]. Given the excellent rationale for the involvement of NTS1 in the pharmacology of psychiatric disorders, there is an obvious lack of molecules that qualify for an investigation of the effects in preclinical and clinical models. In particular, safe and potent molecules suited for CNS applications are needed [14].

Fragment-based screening is a well-established approach in drug discovery to identify novel starting points for chemistry, having resulted in a number of marketed drug molecules [15, 16]. Zelboraf (Vemurafenib) is an example of a drug developed initially from a fragment with a low affinity (IC_50_ > 200 μM) [15]. Initial success of fragment-based screening was demonstrated for kinases and expanded further to a wide range of other protein classes [17]. Originally, the screening of fragment libraries was performed using NMR or X-ray crystallography as initial biophysical method to assess binding [18, 19]. Recently, Surface Plasmon Resonance technology has been used more frequently due to low protein consumption, ability to test several thousands of compounds and to asses both, binding affinity and stoichiometry of binding [20, 21].

The application of SPR technology to characterize the interaction of soluble proteins is well established, whereas it is still limited in the case of analyzing ligand binding to membrane proteins (MPs) [21, 22]. The main bottleneck while working with MPs is their low expression levels and their inherent instability in a non-native, non-membranous environment, and thus the challenge in immobilizing correctly folded MPs [23–25].

Pioneering work on application of SPR technology to detergent-solubilized MPs was presented by Myszka’s group on two chemokine receptors, CCR5 and CXCR4 [26, 27]. Both receptors were solubilized directly from cells with a mixture containing detergents and lipids, and captured on a SPR sensor via a specific antibody without prior purification. The binding data for small molecule ligands analyzed on CCR5 and CXCR4 receptors were a breakthrough in the SPR analysis of MPs [27]. In addition to GPCRs, SPR methods were developed to characterize ligand binding to ion channels, and binding affinity as well as binding kinetics were investigated, *e*.*g*., for the Acid Sensing Ion Channel 1a [28]. Further studies on isolated membrane receptors subsequently focused on fragment screening applications. Aristotelous *et al*. [29] demonstrated screening of a library containing 656 fragments with molecular weight from 94 to 341 Da on the wild-type β2 adrenergic receptor, revealing interactions in the nanomolar range. In this study β2 adrenergic receptor was solubilized and purified in detergent micelles, and finally immobilized via a C-terminal polyhistidine-tag on a sensor surface.

Most current studies on purified GPCRs have concentrated on using stabilized variants of such receptors and significant progress in biophysical screening (SPR and TINS techniques) on MPs was demonstrated by Heptares [24, 25, 30, 31]. For example, SPR screening of low molecular weight ligands was shown for stabilized β1-adrenergic and A_2A_ adenosine receptors captured via a His-tag on the sensor surface [24, 25, 30]. Recently, the discovery of dual inhibitors for orexin receptors (OX_1_ and OX_2_) was reported [32]. To date all stabilized GPCRs applied in SPR-based fragment screening were developed by iterative single point mutagenesis, and mostly by alanine scanning.[24, 25, 30, 31] However, directed molecular evolution represents an alternative approach to stabilize MPs [33, 34].

Besides SPR, Nuclear Magnetic Resonance (NMR) represents an orthogonal biophysical method to investigate molecular interactions in drug discovery [35]. The intrinsic feature of NMR to detect target-ligand interactions from nanomolar to millimolar with high sensitivity is of fundamental advantage, particularly for the application of fragment-based screening. In combination with the underlying versatility of NMR spectroscopy, several experimental settings were developed, resulting in strong contributions to fragment-based lead discovery [36–40]. For the screening of larger libraries, a ligand screening method called TINS (Target Immobilized NMR Screening) has been developed which was successfully applied to soluble as well as membrane proteins [41], and even to thermostabilized GPCR targets [25, 42].

In this study, we used the neurotensin receptor 1 stabilized by directed molecular evolution to discover novel lead molecules by fragment-based screening that qualify for further optimization by medicinal chemistry efforts. NTS1 receptor represents a challenging target for identification of small molecules due to the binding site tailored for its endogenous peptidic ligand. Nevertheless, we present here for the first time a SPR-based fragment screen of a peptide-binding GPCR, in combination with NMR, to identify, validate and subsequently characterize hit molecules. *In silico* analysis of the fragments binding to the NTS1 receptor based on the known X-ray structures suggests unique avenues for medicinal chemistry to develop novel small molecule based agonists and antagonists for the NTS1 receptor.

## Results

### Capturing, binding activity and stability of NTS1-H4 receptor

**Fig 1:**
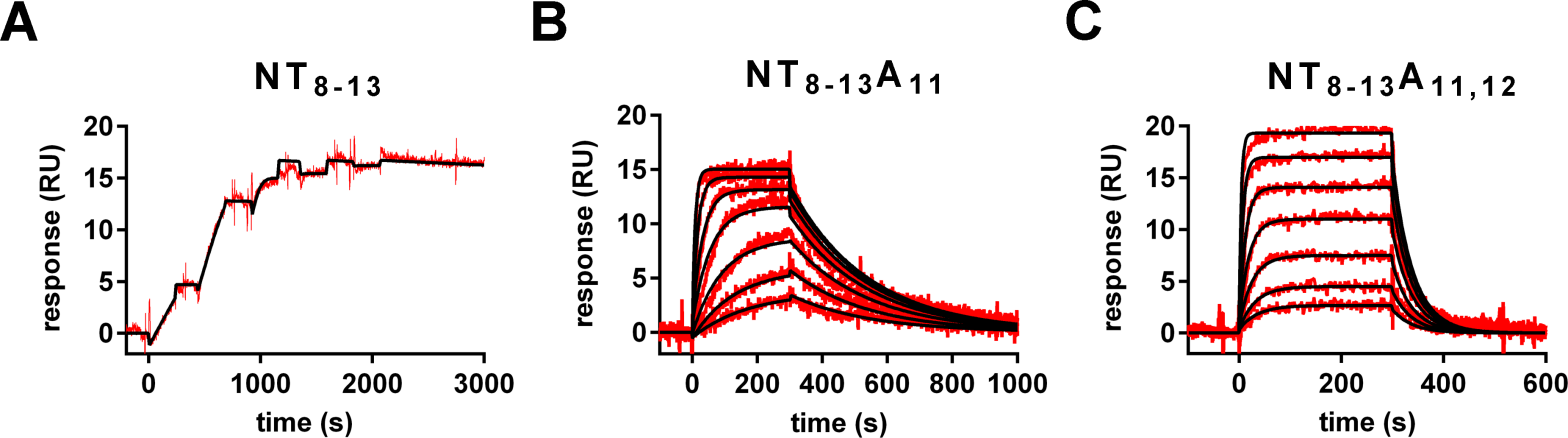
Overlay of binding curves (red) monitored by titration experiments of neurotensin peptides (NT_8-13_, NT_8-13_A_11_, and NT_8-13_A_11,12_) on the NTS1-H4 surface with low receptor density and mathematically calculated curves for a one-to-one interaction binding model (black). (A) Binding curves for neurotensin peptides NT_8-13_ titrated up to 25 nM in a single cycle kinetic experiment (dilution factor 2); (B) and (C) Binding curves for NT_8-13_A_11_, and NT_8-13_A_11,12_ titrated up to 100 or 500 nM in multiple cycle kinetic experiments (dilution factor 2), respectively.

The receptor was expressed in *E*. *coli* with a *C*-terminal avi-tag located remote from the binding site, which is directly biotinylated and allows immobilization on streptavidin-coated chips. Purified NTS1-H4 receptor was successfully captured at high densities (8500–9500 RUs) on the streptavidin-coated SPR sensor with high reproducibility within single experiments as summarized in S1 Fig. Binding activity of captured NTS1-H4 receptor was validated with three peptides derived from neurotensin, which is the endogenous agonist of the NTS1, and one non-peptidic small molecule antagonist SR142948 (Fig 1) and S1, S2 and S4 Figs [8, 34]. As summarized in Table 1, the neurotensin peptides differ in affinity by a factor of 72 (NT_8-13_A_11_) and 906 (NT_8-13_A_11_,_12_) compared to the truncated wild type neurotensin, NT_8-13_ and thus are suited ligands for SPR assay development. These results confirm earlier reports of activities with mutated NT peptides [43]. The NTS1 receptor antagonist SR142948 occupies the same binding site as the neurotensin peptide, as demonstrated in a competition assay with NTS1-H4 receptor and its peptide agonist (see S3 Fig for more details). Again, these results confirm reported data [4] and, as summarized in Table 1 all binding data of the ligands correlate well to earlier publications. Consequently, the SPR method established in this report is well validated and able to accurate binding measurements. In addition, the NTS1-H4 receptor reveals long-term stability when captured on the SPR sensor with the reference peptide NT_8-13_A_11_. We observed only negligible reduction of SPR signal by 2% monitored for NT_8-13_A_11_ within 24 hours as shown in S4 Fig enabling high accuracy and sensitivity of the measurements as a prerequisite for successful fragment binding experiments.

**Table 1:** SPR binding parameters (calculated from triplicate measurements) of agonistic (peptides) and antagonistic (small molecule) ligands for neurotensin receptor 1 monitored on rat NTS1-H4 receptor surface and published *in vitro*/*in vivo* data for rat and human NTS1 receptor.

Ligand	Sequence	MW (Da)	SPR (rat NTS1-H4 receptor)			published *in vitro*/*in vivo* data for rat and human(*) NTS1 receptor (EC_50_, IC_50_, K_i_, K_D_ ± σ (M))	
			k_on_ ± σ (M^-1^s^-1^)	k_off_ ± σ (s^-1^)	K_D_ ± σ (M)	NTS1-H4	NTS1 wt
NT_8-13_	RRPYIL	817.0	2.6 ± 1.9 10^6^	5.0 ± 2.9 10^−5^	3.2 ± 2.4 10^−11^	3.4 ± 0.9 10^−10^ (K_i[34]_)	1.6 ± 0.1 10^−10^ (K_D[51]_) 1.4 ± 0.1 10^−10^ (K_D[51]_)*
NT_8-13_A_11_	RRPAIL	724.9	2.2 ± 0.3 10^6^	5.2 ± 1.1 10^−3^	2.3 ± 0.4 10^−9^	**-**	**-**
NT_8-13_A_11,12_	RRPAAL	682.8	1.2 ± 0.4 10^6^	3.5 ± 0.8 10^−2^	2.9 ± 0.6 10^−8^	**-**	**-**
SR142948	-	685.9	4.0 ± 1.2 10^6^	1.5 ± 0.4 10^−3^	4.0 ± 1.4 10^−10^	5.0 ± 1.0 10^−10^ (K_i[34]_)	8.4 ± 0.9 10^−9^ (IC_50[4]_)

### SPR Fragment Screening on NTS1-H4 and SPR hit confirmation

**Fig 2:**
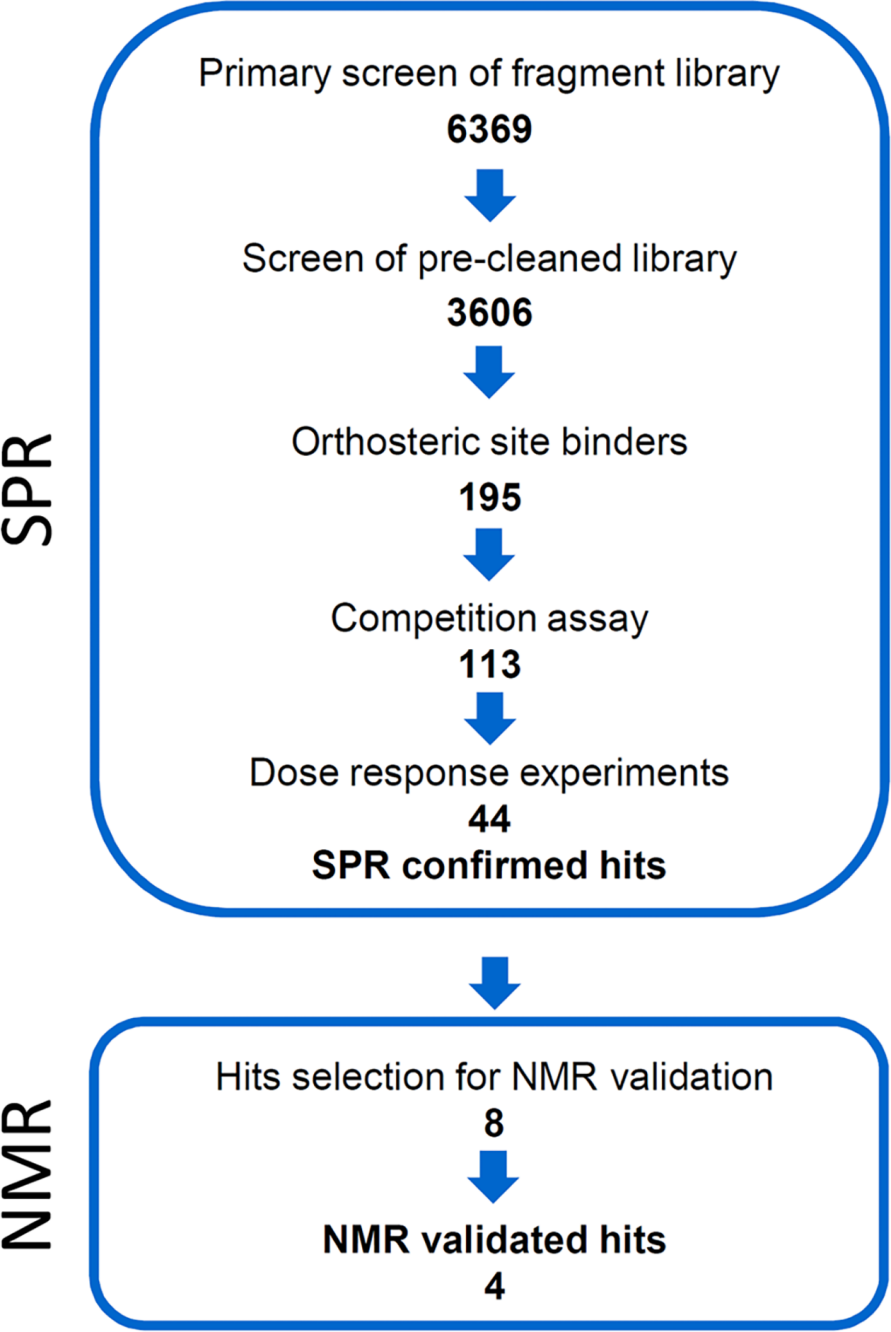
SPR screening workflow of the Roche fragment library on the thermostabilized NTS1 receptor NTS1-H4 and subsequent hit validation by NMR.

**Fig 3:**
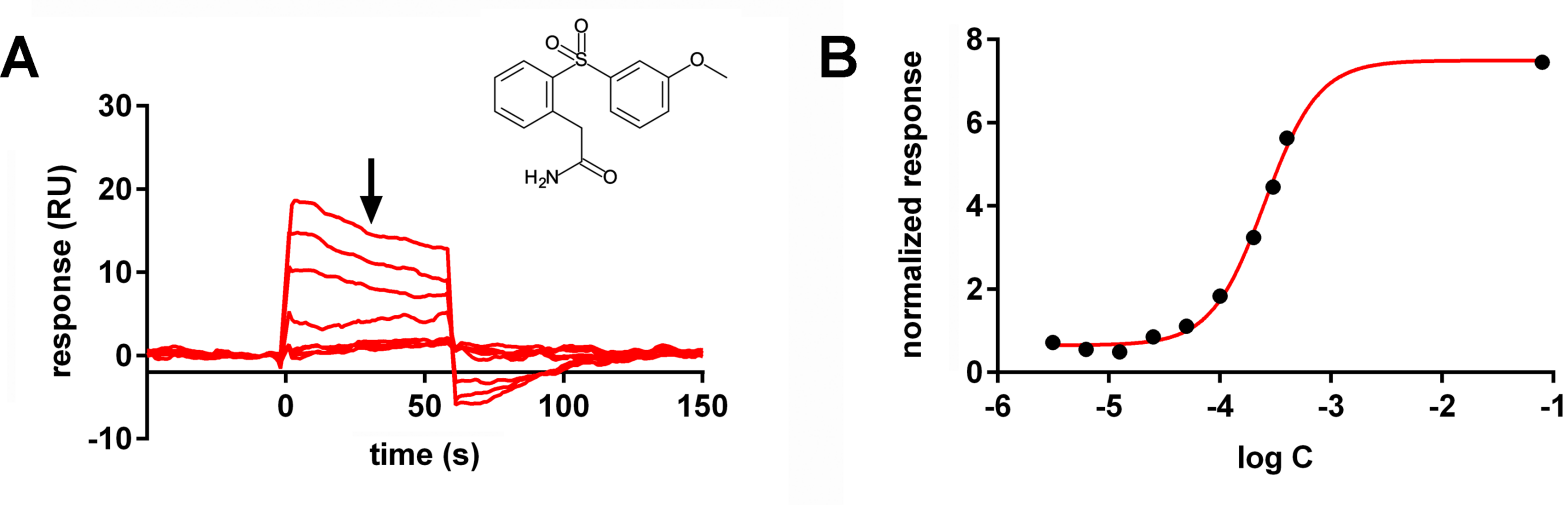
SPR binding data for fragment 2 measured with purified NTS1-H4 receptor. (A) SPR binding curves monitored in titration experiment up to a fragment concentration of 400 μM (3.13, 6.25, 12.5, 25.0, 50.0, 100, 200, 300 and 400 μM). (B) Dose-response plot of SPR signal. Black dots depict the amplitude of resonance signals in the middle of the association phase (indicated by arrow in A). Resonance signals are normalized regarding molecular mass and fitted to one-to-one interaction model with a fixed maximal response (empty dot) determined by the positive control (NT_8-13_A_11_) at saturating concentration.

The Roche fragment library contains 6369 molecules, with 99.7% of all fragments having molecular masses smaller than 350 Da (see S6 Fig for more details) [20]. Upon screening a first fragment series, we observed rapid reduction of the binding activity of captured NTS1-H4 receptor. Consequently, we decided to perform a pre-cleaning of the fragment library to exclude promiscuous binders. Applying such a pre-cleaning of the Roche fragment library on a NTS1-H4-coated surface, 2763 fragments were removed. Examples for promiscuous fragments are shown in S7 Fig. Furthermore, we established a reference channel for NTS1-H4 receptor by “blocking” the receptor binding site with the agonist, using a truncated neurotensin NT_8-13_ peptide, which has a very slow dissociation rate constant, to differentiate between orthosteric and non-orthosteric binding (Table 1). In such an assay set-up, we have monitored virtually no binding of the reference peptide NT_8-13_A_11_ to the reference protein channel (S4B Fig). By screening of the pre-cleaned library of 3606 fragments, 195 fragments have been selected as hits which demonstrated selective binding to the orthosteric binding site of NTS1 receptor. 113 of 195 hits showed competition with a peptidic agonist, the double mutated and truncated neurotensin NT_8-13_A_11,12_. Finally, we confirmed 44 hits out of 113 hits in dose-response experiments (hit rate of 0.69%) with affinities in the range of 18 to 441 μM (Fig 2 and Fig 3).

### Hit validation by NMR as orthogonal biophysical method

**Fig 4:**
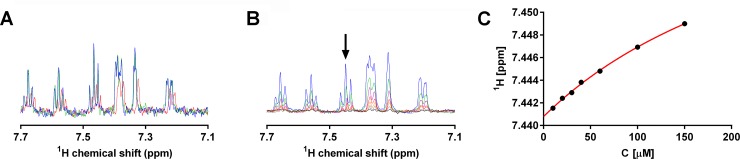
NMR control experiments and binding data for fragment 2 measured with purified NTS1-H4 receptor. The affinity of fragments for L-MNG detergent used for the solubilization of NTS1-H4 was tested in preliminary experiments. The 1D ^1^H aromatic spectrum of 10 μM fragment is shown in buffer without L-MNG detergent (green), in buffer with 0.01% L-MNG (blue), and in buffer with 0.01% L-MNG and 5 μM NTS1-H4 (red). All tested fragments showed no affinity for L-MNG detergent micelles, and the observed line broadening effect upon protein addition indeed originates from the interaction between fragment and NTS1-H4. (B) Overlay of fragment 2 1D ^1^H NMR spectra that reflect a titration series up to 150 μM (10 (black), 20 (grey), 30 (orange), 40 (red), 60 (cyan), 100 (green) and 150 (blue) μM). (C) The interactive curve fitting program XLfit was used to determine K_D_ values from subtle chemical shift differences observed in 1D ^1^H NMR spectra.

**Fig 5:**
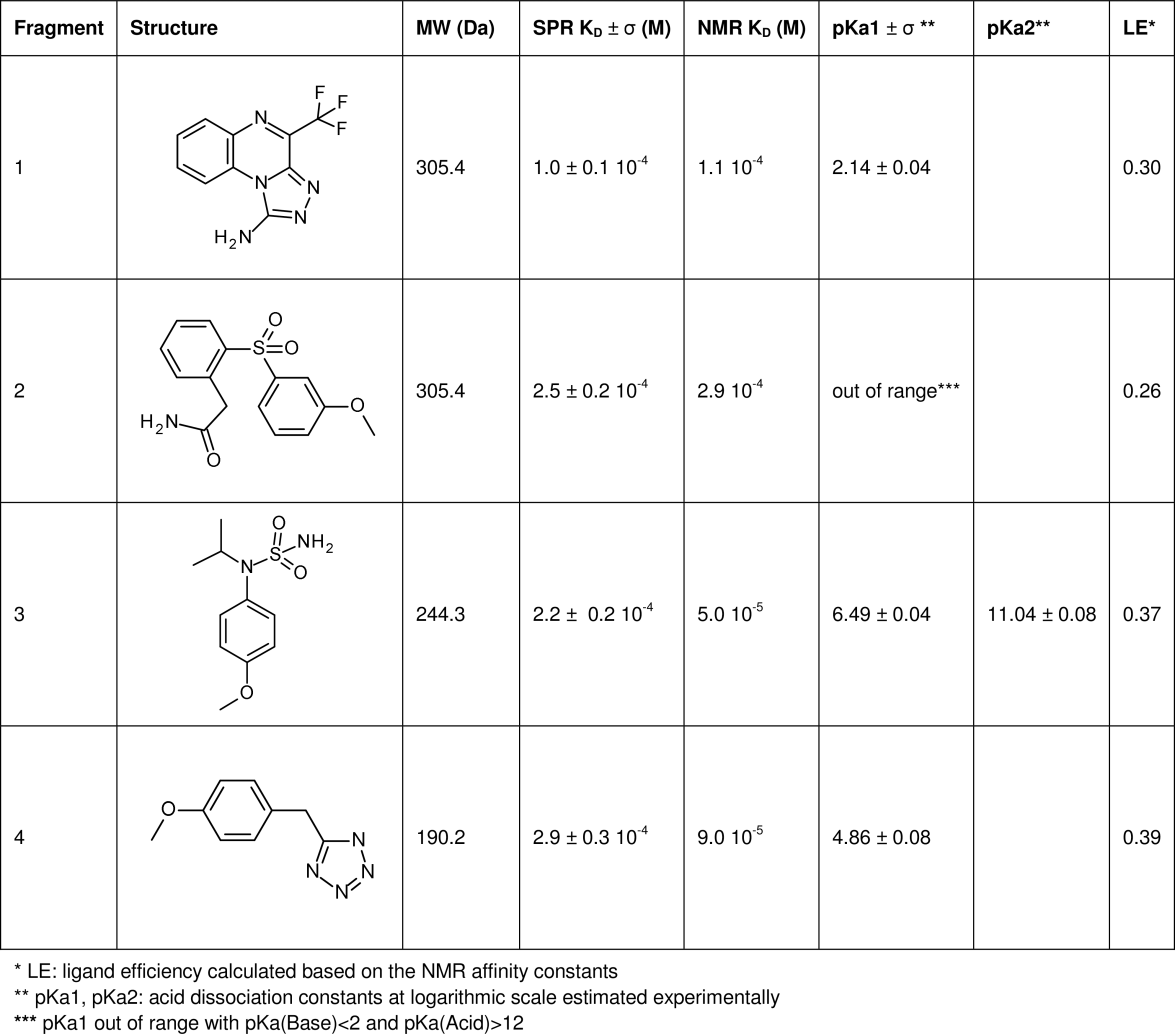
SPR binding parameters (calculated from triplicate measurements) of agonistic (peptides) and antagonistic (small molecule) ligands for neurotensin receptor 1 monitored on rat NTS1-H4 receptor surface and published *in vitro*/*in vivo* data for rat and human NTS1 receptor.

Ligand-observed proton-detected titration experiments by NMR were performed to validate SPR fragment hits. The schematic view of S8 Fig shows that of the 13 identified compound clusters, 8 fragment hits from 4 clusters and one singleton were selected. The selection was based on compound availability and aqueous solubility. Selected fragment hits were first analyzed in buffer containing L-MNG alone to investigate their potential undesired interaction with detergent micelles. In these "blank" experiments neither effects on the chemical shifts nor line broadening of fragment resonance signals were observed in 1D ^1^H spectra (Fig 4A). In contrast, the presence of solubilized NTS1-H4 caused a significant loss in signal amplitudes of up to 65% at a fragment-to-protein ratio of one-to-one, indicating binding (Fig 4A). In addition, subtle changes of ^1^H chemical shifts were observed in 1D titration experiments (Fig 4B and 4C). These chemical shift perturbations clearly indicate specific binding of fragments to NTS1-H4. As a result, the binding of 4 out of 8 SPR-confirmed fragment hits were validated by NMR. The other 4 fragments could not be validated by NMR. For the calculation of the dissociation constants between fragments and NTS1-H4 receptor ^1^H chemical shifts of aromatic signals were used. K_D_ values were determined for the 4 validated binders in the range from 50 to 300 μM (Fig 5).

### Computational analysis of the fragment hits

The 44 SPR-confirmed hits were analyzed according to their chemical similarity, resulting in 13 clusters and 9 singletons. Furthermore, computational investigations were undertaken for the 4 NMR-validated hits, which were assessed as chemically tractable and thus warranting a more thorough exploration of their binding mode.

**Fig 6:**
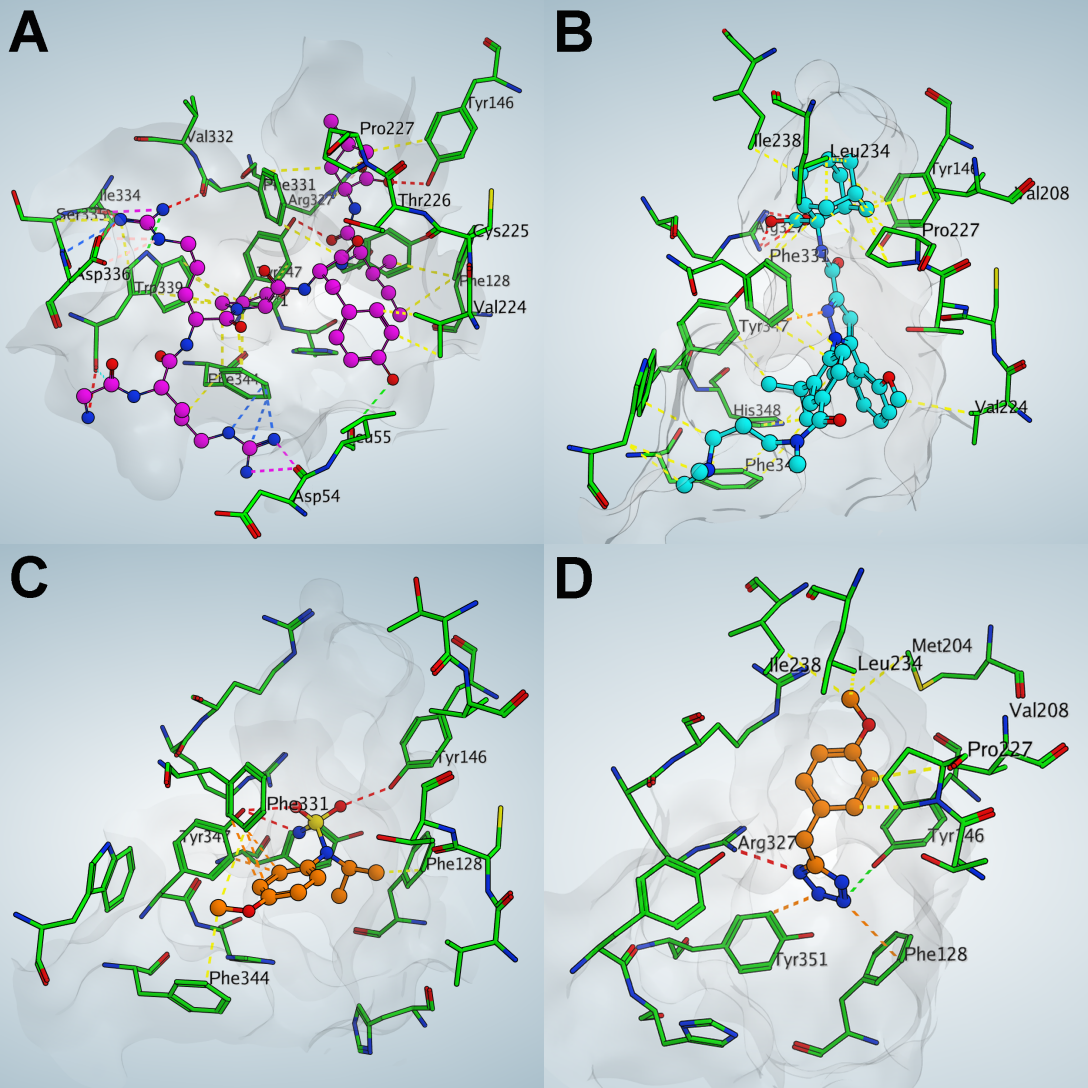
Comparison of the experimental X-ray structure complex of the peptidic agonist (NT)_8-13_ with the antagonist SR142948 as well as fragment structures as derived from docking experiments. (A) The binding mode of the peptidic agonist (X-ray structure [4] shown as ball and stick, with carbon atoms colored in pink) in NTS1–H4 (shown as stick, with carbon atoms colored in green) binding pocket (shown as the molecular surface colored as white). Nitrogen atoms are colored in blue, and oxygen atoms are colored in red in both, ligand and protein. Yellow, red and orange dash lines represent hydrophobic, electrostatic, and **π**-**π** interactions, respectively. The same rules are applied for the following figures. (B) The binding mode of the SR142948 antagonist (docked conformation shown as ball and stick, with carbon atoms colored in cyan) in NTS1 binding pocket.(C) The binding mode of fragment hit 2 (docked conformation shown as ball and stick, with carbon atoms colored in orange) in NTS1 binding pocket. (D) The binding mode of fragment hit 4 (docked conformation shown as ball and stick, with carbon atoms colored in orange) in NTS1 binding pocket.

To direct synthesis of novel compounds and facilitate a more rational design of sublibraries for the medicinal chemistry we docked the antagonist SR142948 and 4 fragment hits into the binding pocket of the NTS1-H4 receptor to analyze their pharmacophore similarities. The docking was guided by the X-ray structures of the peptide agonist bound complex structures as published at high resolution (PDB entry 3ZEV, 4BUO, 4BV0 and 4BWB) (Fig 6A). Analysis and comparison of shared functionalities, conformational restrains and space requirements between the peptide agonist and the antagonist SR142948 helped to pick the most likely docking pose of the antagonist in the receptor binding site. The antagonist SR142948 covers the entire binding site of the NTS1-H4 receptor similar as compared to the peptide agonist (Fig 6B), with the carboxyl-adamantane moiety anchoring deeply in the hydrophobic cavity of the binding pocket and interacting with the surrounding residues: Tyr146, Val208, Pro227, Leu234, Ile238 and Phe331. As shown in S9A Fig, the negatively charged carboxylic acids of the antagonist SR142948 and the peptide agonist are located in the same receptor binding pocket and pick up the electrostatic interaction with Arg327 of the NTS1-H4 receptor. This interaction seems to be critical for the ligand binding affinity to the NTS1-H4 receptor, as reported previously.[44] The methoxy groups, the phenyl ring and two terminal methyl groups of SR142948 form hydrophobic interaction with the protein. All these interactions could explain the high binding potency of SR142948 to the NTS1-H4 receptor. Although SR142948 interacts with the NTS1-H4 receptor in a similar way compared to the peptide agonist, it doesn’t form the specific interaction with the NTS1-H4 receptor caused by two arginine side chains of the peptide agonist. Here, the backbone of Asp54 on one side and the backbone of Ile334/Ser335 as well as the side chain Asp336 on the other side of the binding area are connected by the peptide ligand. This interaction stabilizes the conformation of the respective protein areas and likely be crucial for agonist functionality. Analysis of the binding modes of the validated fragment hits shows three fragments (fragments 1, 2 and 3) mimicking the aromatic ring of the antagonist SR142948 (Fig 6C, S9B and S9C Fig) and the hydrophobic interaction with the protein. For example, the acetamidephenyl ring of fragment 2 and the 4-methoxy-phenyl moiety of fragment 3 have strong π-π interaction with Phe331. The tetrazole ring of fragment 4 picks negative electrostatic interaction with Arg327, and the methoxy-phenyl ring shows hydrophobic interaction with the protein (Fig 6D). Binding modes of antagonist SR142948 and 4 fragment hits represent possible docking poses which should be confirmed by experimental ligand complex structure determination.

## Discussion

A combination of several key assets contributed to the successful discovery of novel small molecule ligands of the neurotensin receptor 1.

First, a SPR-based fragment screen was set up with a stabilized NTS1 receptor variant selected by a direct molecular evolution, resulting in unique stability even in the absence of a stabilizing ligand. We ensured functionality of the stabilized NTS1-H4 receptor, as demonstrated previously by Egloff *et al*. [4], in a [^35^S]GTPγS signaling assay in which the binding of the agonist neurotensin to the NTS1-H4 receptor triggers exchange of GDP with the non-hydrolysable labeled GTP analog. This assay was carried out with heterotrimeric G proteins composed of Gα_i1_, Gβ_1_, and Gγ_1_.

Second, the NTS1-H4 receptor can bind to agonistic and antagonistic ligands, indicating that conformational flexibility of the receptor to adopt the agonist- or antagonist-bound conformation is maintained [34]. Thus, the NTS1-H4 receptor developed by directed molecular evolution appears to be ideally suited for biochemical and biophysical investigations of ligands with distinct binding modes (*e*.*g*. agonistic and antagonistic binding). This observation correlates well with earlier reported data [34] and enables identification of ligands for NTS1 with different mode of interaction using the very same stabilized protein variant.

Third, the apo-NTS1-H4 receptor variant developed by directed molecular evolution demonstrated high long-term stability when captured on the biosensor and thus facilitated screening of fragments by SPR (S4B Fig).

Fourth, a high density of protein immobilized on the sensor surface achieved high sensitivity of the SPR binding experiment while maintaining biological functionality (no denaturation, accessible binding sites, and active protein conformation). In our experiments, we observed high binding activity of captured NTS1-H4 receptor of around 80% as probed with NT_8-13_A_11_ peptide (S2 and S4 Figs). To assess the binding capability for ligands of NTS1-H4 receptor captured on the biosensor, we performed binding studies with known NTS1 ligands, namely truncated neurotensin peptide and its mutated derivatives, covering a wide range of affinities. In addition, the antagonist SR142948 exhibits binding to NTS1-H4 receptor in the nano- to picomolar range. Table 1 shows a comparison of our measured binding data with published data for NTS1 receptor. Exemplarily, the inhibition constant observed in a competition assay with NTS1-H4-coated beads and fluorescently labeled NT_8-13_ for the antagonist SR142948 (K_i_ of 0.5 nM) correlates well with the affinity constant published in literature and analyzed by SPR (K_D_ of 0.4 nM) [34]. Other SPR binding data for agonistic peptides are comparable with *in vitro* and *in vivo* binding data reported for NTS1 [4, 34, 45, 46]. From these results we conclude that the captured NTS1-H4 receptor demonstrates comparable binding capability as the wild-type neurotensin receptor 1.

Fifth, the use of a well-designed fragment library with over 6000 chemical entities and the establishment of a powerful compound selection strategy, including a compound pre-screening, contributed to the discovery of novel NTS1 ligands. Screening of fragment libraries at high micromolar fragment concentration often results in a relatively high rate of false positives. Nonspecific binding of fragments can be either allocated to the protein surface itself, particularly the transmembrane part, or to interaction of the fragments with the protein-bound lipid and detergent molecules. To select compounds that demonstrate selective binding to the intended receptor ligand binding pocket (the orthosteric binding site), and to reduce false positive hits, it is indispensable to establish a proper reference protein channel, ideally by immobilizing the very same receptor with a blocked ligand binding pocket. Consequently, binding data collected simultaneously on both protein channels (binding site accessible and binding site blocked) allows differentiation between specific binding to the orthosteric receptor binding site and binding to other sites or even non-specific interactions. Such an assay set-up is commonly used in fragment screening with soluble proteins [21, 47]. For example, Perspicace *et al*. [21] have screened a fragment library with chymase protein in the form of the active enzyme and its zymogen (inactive form of the active protein). In the case of fragment screening on DPPIV (dipeptidylpeptidase 4) protein the reference channel was established by modification of immobilized DPPIV protein with an irreversible covalent inhibitor [47].

In summary, by a combination of fragment library pre-cleaning and verification of hit selectivity on a suitable protein reference channel leading we significantly reducted the number of false positive hits. As demonstrated here here, ligands exhibiting a slow dissociation rate (long residence time) or showing even covalent irreversible binding are a straightforward and elegant approach to block the receptor binding site and thus create an almost ideal reference channel, differing from the sample channel by the availability of the binding site. Creation of a receptor mutant that blocks the ligand binding site is an alternative approach. Here, expression, purification as well as assessment of the mutant with respect to binding capabilities for reference compounds need to be established before the screening effort. Another possibility to establish a reference channel would be to screen for selective fragments on the target protein and related receptor types in parallel. For example, the Heptares fragment library was screened on a β_1_-adrenergic (β_1_-AR) mutant receptor in parallel with an adenosine A_2A_ mutant receptor [24, 30]. In such a tandem screen, selective binders were found for both, the thermostabilized receptor β_1_-AR and A_2A_ [24]. In order to ensure a successful outcome of such a tandem fragment screening approach, protein binding activity and protein long-term stability of both receptors need to be preserved under the same experimental conditions (detergents, buffers, temperature etc.), which has been highly challenging to be achieved for membrane proteins in general, and GPCRs in particular. Nonetheless, receptor stabilization through directed evolution has proven to be a very robust strategy for this purpose.

To further increase the confidence into SPR-confirmed hits, 8 selected fragments were validated with an orthogonal biophysical method. Our experiments prove that NMR is a valuable complement to verify the interaction of fragments with unlabeled NTS1-H4 after successful identification in a SPR screening effort. The major disadvantage of NMR, the high consumption of protein compared to other techniques, could be overcome in our studies by the use of a 1.7 mm TCI MicroCryoProbe system. Thus, consumption of NTS1-H4 was reduced to 15 μg per experiment, compared to about 65 μg using a traditional 3 mm cryo probe. The economic use of protein is of crucial importance especially for GPCRs where protein supply is often limited.

To validate the interaction between fragments and NTS1-H4 in low volumes and at low concentrations, 1D ^1^H ROESY experiments were the method of choice, offering a fast and sensitive experimental setup. Due to the ROESY spin-lock, broader NTS1-4H resonance signals do not contribute to the 1D ^1^H spectrum and thus potential overlaps with fragment-hit-derived signals were avoided. In the “blank” experiments with buffer containing L-MNG only, no interaction between fragments and L-MNG micelles was found. Consequently, perturbations of fragment line shape and resonance frequencies were unequivocally assigned to the specific interaction between a respective fragment hit and NTS1. This led to the validation of 4 fragments originating from 3 different chemical clusters and thus encourages further computational work.

Analysis of fragment binding modes by computational methods highlights the great opportunities for synthetic chemistry to further optimize potency and properties of the compounds identified in this study. Exemplarily, possible modifications of fragment 3 could be a replacement of the isopropyl moiety with a substituted phenyl ring or an elongation of the sulfonamine group with modified alkyl chains. Fragment 1 mimics both the 2,6-dimethoxy-phenyl and imidazole rings in the central part of the antagonist structure. This molecule could now be elongated by the modification of a condensed phenyl ring or by substitution of nitrogen in the position 2 of the condensed triazole ring. The negative charge of fragment 4 might limit brain penetration and thus needs attention in further exploration of related compounds.

High ligand efficiencies (LEs) of the fragment hits (Fig 5) support further chemical optimization towards development of novel compounds and potential drug candidates targeting NTS1. However, LE values of fragment hits should be considered only as preliminary guideline due to the expected high error rates for the determination of corresponding K_D_ values at this early stage of the project.

The fragments identified in this work demand a chemistry program that explores their value further. Structural studies of NTS1 receptor complexed with fragment hits will ultimately facilitate the understanding of the binding properties and thus deliver decisive information for a truly efficient optimization of the identified compounds.

## Materials and methods

Cloning of the GPCR expression construct, NTS_1_-H4 protein expression and protein purification are described in the supporting information.

### Surface plasmon resonance binding assays with NTS1-H4 receptor

All SPR binding experiments were performed on Biacore® 3000, T200 and Biacore 4000 (GE Healthcare, Uppsala, Sweden) instruments at 15°C in a running buffer composed of 20 mM Tris, 150 mM NaCl, 0.01% (v/v) L-MNG, 2% (v/v) DMSO, pH 8.0, at flow of 30 μl·min^-1^. Running buffer was prepared freshly every day and filtered with Express™Plus Steritop filters with 0.22 μm cut off (Millipore, Billerica, MA, USA) and degassed prior to SPR analysis. Dose-response experiments for peptides were performed in the buffer without DMSO, as the analyzed peptide concentration range was in nM range and it was not necessary to work with DMSO in the running buffer to correct for the resonance signals.

### Ligands for NTS1-H4 receptor

Peptide ligands (NT_8-13_, NT_8-13_A_11_ and NT_8-13_A_11,12_) were obtained from JPT Peptide Technologies (Berlin, Germany) and the antagonist SR142948 was purchased from Sigma-Aldrich (Buchs, SG, Switzerland). The fragments library was obtained from Roche (Basel, Switzerland).

### Capturing of NTS1-H4 receptor

NTS1-H4 receptor was captured via its biotin-carrying tag on streptavidin pre-coated SA sensors or, alternatively, on streptavidin attached to the sensor via DNA/DNA hybridization (Biotin CAPture kit) (GE Healthcare, Uppsala, Sweden: BR-1005-31 and BR-1000-32 for Biacore 4000, T200 and 2000, respectively; Biotin CAPture kit: 28920233). First, the streptavidin SA sensor was conditioned with 3 consecutive 1-min injections of high salt solution (50 mM NaOH in 1 M NaCl). Next, NTS1-H4 receptor was diluted in running buffer 24-fold from its stock solution to 1 μM and applied twice (2 x 10 min) over the streptavidin sensor surface to achieve relatively high immobilization levels of NTS1-H4. Finally, free biotin solution (1 μM) in running buffer was injected once (1 x 1 min) over the sensor surface to block remaining binding sites in streptavidin. NTS1-H4 receptor was immobilized freshly every day during the entire fragment library screening.

### NTS1-H4 receptor binding activity and stability tests

Binding activity of immobilized NTS1-H4 receptor was tested with three peptides derived from the natural agonist neurotensin; the C-terminal fragment of neurotensin peptide (NT_8-_13; amino acid sequence: RRPYIL), its single Y_11_ to A mutant (NT_8-13_A_11_; amino acid sequence: RRPAIL) and its double mutant Y_11_ to A and I_12_ to A (NT_8-13_A_11,12_; amino acid sequence: RRPAAL) (Table 1). The singly mutated peptide NT_8-13_A_11_ was used at 100 nM as a positive control for fragment screening to test binding activity and stability of NTS1-H4 receptor, while the doubly mutated peptide NT_8-13_A_11,12_ was applied in the competition experiments with fragment hits. Kinetic experiments with peptidic agonists and an antagonist were performed at least in triplicates at the low density of NTS1-H4 receptor (CAP kit) at flow rate of 30 and 50 μl·min^-1^.

### Fragment-based screen with NTS1-H4 receptor

A fragment-based screen with stabilized NTS1-H4 receptor was performed on the Biacore 4000 (GE Healthcare, Uppsala, Sweden) instrument. 6369 compounds available out of the 6611 from the Roche Fragment Library were screened at a concentration of 100 μM. NTS1-H4 receptor was immobilized on the streptavidin surface of a SA sensor as described above on spot 1 and 2 in four flow channels in parallel on the same SA sensor. After receptor immobilization the sensor surface was contacted with a 1 μM biotin solution to saturate remaining free binding sites in streptavidin on the sensor. Spots 4 or 5 with blocked streptavidin were not modified with any other protein and were used as additional “empty” reference surfaces during the fragment screen to monitor possible nonspecific binding of screened compounds on the sensor surface.

#### Blocking of immobilized NTS1-H4 receptor to create protein reference surface

The NTS1-H4 receptor surface was contacted 6 min with the wild-type NT_8-13_ peptide at a concentration of 100 nM in one of the two spots in every channel on the SA sensor. Blocking of the NTS1-H4 receptor during screening of the fragment library was repeated within the screening procedure to assure full blocking of the receptor in the reference spot.

#### Sample preparation for primary and secondary screening

Samples were obtained as 5 mM DMSO stock solutions in a 96-well plate format. First, 8 microliters of each sample was split in three 2 microliter replicates into new 96-well plates using a CyBi®-Well instrument equipped with a 96-fold pipetting head. Finally, DMSO solutions were diluted with Tris running buffer excluding DMSO to achieve finally 100 μM fragment concentration and 2% (v/v) DMSO content.

#### Primary screen/cleaning of the fragment library

First, the primary screen of the Roche fragment library was performed at 100 μM in order to clean the library from those fragments which demonstrate promiscuous behavior and would affect NTS1-H4 receptor binding activity. Binding of fragments to the immobilized NTS1-H4 receptor was monitored after 60 s in the association phase as well 60 s in dissociation phase. Additional injection of the running buffer (“carry-over” control) after binding of every sample was performed directly after every fragment binding. Selection of the “typically” behaving compounds was based on the report points’ analysis. Four report points were set in the binding curves in the association (report point: binding early, binding late, 5 s after start and before end of an association phase, respectively) and dissociation phase (report points: dissociation early and dissociation late; 5 and 60 s after end of an association phase, respectively). Selected fragments were screened further in the secondary (main) screen. Fragment samples were prepared analogously as in the primary screen.

#### Hit selection

Hits analyzed in the secondary (main) screening which demonstrated selective binding to the NTS1-H4 receptor and no binding to the blocked NTS1-H4 receptor were selected as positive hits for analysis in the competition experiments. Double-referenced signals of fragment hits and positive controls were normalized regarding molecular mass, and normalized to the signal of the positive control. Fragments showing a selective binding signal which was higher than three times the standard deviation of the negative control (buffer) (>3 x σ) and was in the range of stoichiometric binding (signal lower than 1.3 fold of normalized signal for the positive control) were selected for competition experiments.

#### Competition experiments

Hits selected as orthosteric site binders were characterized in competition assays with doubly mutated C-terminal neurotensin peptide NT_8-13_A_11,12_. Peptide NT_8-13_A_11,12_ and fragment hits were analyzed separately at 500 nM and 100 μM, respectively, and as a mixture using the same concentrations (500 nM peptide, 100 μM fragment) in running buffer supplemented with 2% DMSO (v/v). Samples of mixtures were prepared manually.

#### Dose-response experiments

Hits confirmed in the competition experiments with doubly mutated peptide NT_8-13_A_11,12_ were analyzed in dose-response experiments. Powders of fragments were first dissolved in DMSO to obtain 10 or 100 mM DMSO stock solutions and further titrated in the running buffer up to 500 μM. Dose-response series of fragments were prepared with the pipetting robot Genesis RSP 100 (Tecan, Switzerland, Männedorf).

### SPR data processing

All SPR data processing and analyses were performed using BiaEvaluation Software (version 1.0 (Biacore 4000) and 4.1 (Biacore 3000)), and GraphPadPrism (version 6.04). All monitored binding resonance signals were corrected regarding DMSO solvent signals and further double-referenced, *i*. *e*., signals monitored on the binding active channel were subtracted by signals from the reference channel (sensor surface not modified with any reference protein) and by buffer signals. For kinetic evaluation, data were globally fit to the mathematical binding model describing a one-to-one interaction. For equilibrium analyses, the SPR signals at equilibrium were plotted against analyte concentration and fit to the one-to-one interaction model with four parameters.

### Nuclear magnetic resonance as orthogonal method for hit validation

For titration experiments 34 μl of 20 μM unlabeled NTS1 in 10 mM HEPES pH 8, 150 mM NaCl, 0.01% (w/v) L-MNG and 12% D_2_O were transferred into a disposable 1.7 mm NMR tube. Fragment compounds were titrated gradually to the NMR sample which was thoroughly mixed and quickly centrifuged briefly to collect the solution at the bottom of the tube. Importantly, fragments were titrated from aqueous stock solutions containing 30% DMSO acting as solubilizer. Thereby the final DMSO concentration could be kept below 3% after seven titration steps. Tested fragment concentrations were 10 μM, 20 μM, 30 μM, 40 μM, 60 μM, 100 μM and 150 μM.

All NMR experiments were performed on a Bruker Avance 500 MHz high resolution spectrometer equipped with a 1.7 mm TCI MicroCryoProbe at a temperature of 300 K. Spectrometer operation and data processing was done by the use of Topspin 2.1 (Bruker, Fällanden). In all spectra the water signal was suppressed by a 50 Hz pre-saturation employed during the interscan relaxation delay. This relaxation delay was set to 3 s. The interaction of NTS1 with selected fragments was followed by 1D ^1^H ROESY experiments using a mixing time of 100 ms, 256 scans, a sweep width of 31 ppm and the acquisition time of 2 s.

Polynomial baseline correction was employed as needed and chemical shifts were extracted manually. K_D_ values from chemical shift perturbations were calculated by XLfit using the equation for very fast exchange [48].

### Molecular docking

The molecular docking of antagonist and fragment hits in NTS1 binding pocket was carried out by Gold (Version 5.3) with default parameters.

### Purity control and synthesis of NMR validated hits

Non deuterated DMSO solutions of validated hits were analyzed by HPLC(UV)-MS and NMR in order to determine the purity of the compounds.

High-performance liquid chromatography coupled to mass spectrometry (HPLC−MS) was performed with an Agilent 6520 QTOF LCMS system connected to an Agilent 1290 LC, equipped with a Zorbax Eclipse Plus C18 column (50 mm ×2.1 mm, particle size 1.8 μm). At a flow rate of 0.8 ml/min a stepwise gradient of Water (+ 0.01% HCOOH) (Eluent A) to Acetonitrile (+ 0.01% HCOOH) (Eluent B) was employed as follows: 0 min 5% B; 0.3 min 5% B; 4.5 min 99% B; 5 min 99% B; 5.1 min 5% B. Mass spectra were acquired in both ESI+ and ESI− mode, scanning from m/z 100 to 3200 Da and UV detection was done at 215 (or 220) and 265 (or 255) nm.

NMR Spectra were recorded on a Bruker Avance III 600 MHz spectrometer, equipped with a 5mm TCI Probe, at 25°C. Samples were diluted with DMSO-d6 to an end volume of 160 μl to be measured in 3mm NMR tubes. Double pre-saturation on the water and DMSO resonance frequency as well as ^13^C decoupling were employed in a gradient ^1^H-NMR experiment.

The purity level of the compounds was >95% as determined by both methods.

The syntheses of fragments 1 and 4 were described.[49, 50] The syntheses of fragments 2 and 3 have not been disclosed to the public.

## Supporting information

S1 Fig Capturing of NTS1-H4, biotinylated at the C-terminal avi-tag, on the biosensor overlay of 8 sensorgrams monitored on a Biacore A4000® during the capturing of the NTS1-H4 receptor on streptavidin pre-coated SA sensor. The resonance signal was monitored on eight spots in four flow channels (two spots per flow channel) in parallel. NTS1-H4 receptor was contacted twice (twice 20 min at a receptor concentration of 1 μM) with the sensor surface to achieve protein densities of ~9000 RUs. Finally, a biotin solution at 500 μM was injected over the sensor surface to block remaining free binding sites on streptavidin.
(PDF)

S2 Fig Binding of NT_8-13_A_11,12_ to NTS1-H4. **(**A) dose-response titration of NT_8-13_A_11,12_ over high density NTS1-H4 surface monitored by peptide titration up to 500 nM (dilution factor 2). (B) Sigmoidal dose-response curve and mathematical fit for one-to-one interaction with a maximal signal calculated theoretically. The apparent affinity constant (K_D_) was estimated to be 90 nM.
(PDF)

S3 Fig Competition of neurotensin peptide NT_8-13_ and antagonist SR142948 on the NTS1-H4 receptor. NT_8-13_ agonist (saturating concentration of 100 nM) and antagonist SR142948 (saturating concentration of 100 nM) were injected subsequently over the NTS1-H4 receptor-coated surface. No binding of SR142948 was detected on the NTS1-H4 receptor that had been saturated previously by agonist NT_8-13_ peptide, indicating complete occupancy of the binding site and binding to the same binding site.
(PDF)

S4 Fig Stability of captured NTS1-H4 receptor. (A) Overlay of four binding curves monitored for NT_8-13_A_11_ on a NTS1-H4 receptor-immobilized surface. (B) Stability plot monitored for NTS1-H4 receptor with NT_8-13_A_11_ over 24 hours. Dots in the diagram represent the amplitude of SPR signals observed at the end of the association phase for NT_8-13_A_11_ on the binding active (red filled dots) and blocked (red empty dots) NTS1-H4 receptor surface. Empty black diamonds and empty black squares (superimposing signals) represent signals monitored by buffer injections over active and blocked NTS1-H4 receptor surface, respectively. Blocking of the orthosteric binding site in NTS1-H4 receptor on the reference channel was performed by injection of NT_8-13_ peptide.
(PDF)

S5 Fig Titration of SR142948 antagonist up to 25 nM over the NTS1-H4 surface monitored in single cycle kinetic mode (red curve) overlaid with the calculated curve for a one-to-one interaction (black curve) and structure of the SR142948 antagonist. (PDF)

S6 Fig Distribution of molecular mass of the fragments within the Roche fragment library comprising 6369 structures. 99.7% of fragments in the library have a molecular mass below 350 Da.
(PDF)

S7 Fig Binding curves monitored by the pre-cleaning process of the fragment library. (A) Typical binding curve for a fragment demonstrating fast kinetics (fast association, saturation of signal in the association phase (signal plateau) and fast dissociation, returning to baseline). (B) Promiscuous fragments: stickiness of compound or atypical sensorgrams (signal lowering in the association phase and signal dropping below baseline in the dissociation phases).
(PDF)

S8 Fig Clustering of SPR hits and hit selection for validation by NMR. Among 44 SPR-confirmed hits, 13 clusters and 9 singletons were identified. 8 hits representing 4 clusters and 1 singleton were selected for NMR validation. 4 hits validated by NMR represent 2 clusters and 1 singleton.
(PDF)

S9 Fig The superposition of the peptidic agonist (NT)_8-13_ with the antagonist SR142948 as well as fragment structures as derived from docking experiments. (A) The superposition of the peptidic agonist (X-ray structure [4] shown as ball and stick, with carbon atoms colored in pink) with the antagonist SR142948 (docked conformation shown as ball and stick, with carbon atoms colored in blue) in NTS1-H4 (shown as stick, with carbon atoms colored in green) binding pocket (shown as the molecular surface colored as white). Nitrogen atoms are colored in blue and oxygen atoms are colored in red in both ligand and protein. The same rules are applied for the following figures. (B) The binding mode of fragment hit 1 (docked conformation shown as ball and stick, with carbon atoms colored in orange) in NTS1-H4 binding pocket. Yellow dash line represents hydrophobic interaction, red dash line represents electrostatic interaction, and orange dash line represents **π**-**π** interaction. The same rules are applied for the following figure. (C) The binding mode of fragment hit 3 (docked conformation shown as ball and stick, with carbon atoms colored in orange) in NTS1-H4 binding pocket.
(PDF)

S1 File Supporting information. (DOCX)

## Abbreviations

ASIC1a: acid sensing ion channel 1a
APBS: Adaptive Poisson-Boltzmann Solver
CXCR4: C-X-C chemokine receptor type 4
CCR5: C-C chemokine receptor type 5
CHS: cholesteryl hemisuccinate
DDM: n-dodecyl-β-d-maltoside
DMSO: dimethylsulfoxide
GPCR: G protein-coupled receptor
L-MNG: lauryl maltose neopentyl glycol
MP: membrane protein
NMR: nuclear magnetic resonance
NT: neurotensin
NT_8-13_: *C*-terminal neurotensin fragment
NT_8-13_A_11_: *C*-terminal neurotensin fragment with single mutation
NT_8-13_A_11_,_12_: *C*-terminal neurotensin fragment with double mutation
NTA: nitrilotriacetic acid
NTS1: neurotensin receptor 1
NTS1-H4: biophysically stabilized variant of NTS1, obtained by directed molecular evolution
PDB: Protein Data Bank
SPR: surface plasmon resonance
TINS: target immobilized NMR screening
TM: transmembrane
RU: response unit
wt: wild type
