# Supplementary material for: SPR-based fragment screening with neurotensin receptor 1 generates novel small molecule ligands
Source: PLoS One. 2017 May 16;12(5):e0175842. doi: 10.1371/journal.pone.0175842 (PMC5433701; doi:10.1371/journal.pone.0175842)
Supplement: S1 Fig — The resonance signal was monitored on eight spots in four flow channels (two spots per flow channel) in parallel. NTS1-H4 receptor was contacted twice (twice 20 min at a receptor concentration of 1 μM) with the sensor surface to achieve protein densities of ~9000 RUs. Finally, a biotin solution at 500 μM was injected over the sensor surface to block remaining free binding sites on streptavidin. (PDF) [file pone.0175842.s001.pdf]

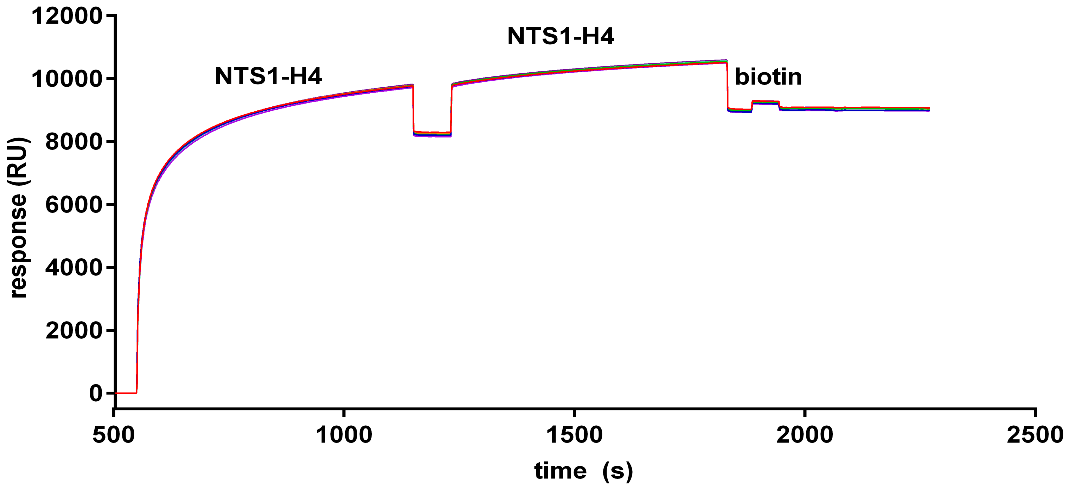

S1 Fig. Capturing of NTS1-H4, biotinylated at the C-terminal avi-tag, on the biosensor overlay of 8 sensorgrams monitored on a Biacore A4000® during the capturing of the NTS1-H4 receptor on streptavidin pre-coated SA sensor. The resonance signal was monitored on eight spots in four flow channels (two spots per flow channel) in parallel. NTS1-H4 receptor was contacted twice (twice 20 min at a receptor concentration of 1 μM) with the sensor surface to achieve protein densities of ~9000 RUs. Finally, a biotin solution at 500 μM was injected over the sensor surface to block remaining free binding sites on streptavidin.
