# Supplementary material for: SPR-based fragment screening with neurotensin receptor 1 generates novel small molecule ligands
Source: PLoS One. 2017 May 16;12(5):e0175842. doi: 10.1371/journal.pone.0175842 (PMC5433701; doi:10.1371/journal.pone.0175842)
Supplement: S2 Fig — (A) dose-response titration of NT8-13A11,12 over high density NTS1-H4 surface monitored by peptide titration up to 500 nM (dilution factor 2). (B) Sigmoidal dose-response curve and mathematical fit for one-to-one interaction with a maximal signal calculated theoretically. The apparent affinity constant (KD) was estimated to be 90 nM. (PDF) [file pone.0175842.s002.pdf]

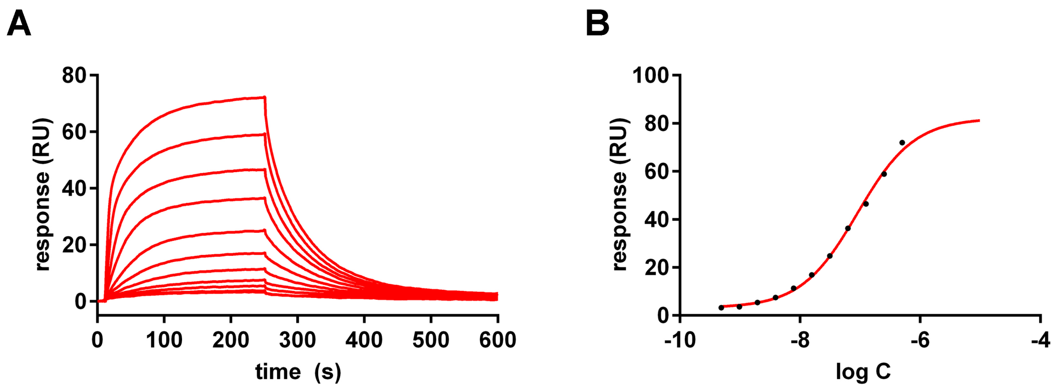

S1 Fig. Binding of $NT_{8\text{-}13}A_{11,12}$ to NTS1-H4. (A) dose-response titration of $NT_{8\text{-}13}A_{11,12}$ over high density NTS1-H4 surface monitored by peptide titration up to 500 nM (dilution factor 2). (B) Sigmoidal dose-response curve and mathematical fit for one-to-one interaction with a maximal signal calculated theoretically. The apparent affinity constant (K_D) was estimated to be 90 nM.
