# Supplementary material for: SPR-based fragment screening with neurotensin receptor 1 generates novel small molecule ligands
Source: PLoS One. 2017 May 16;12(5):e0175842. doi: 10.1371/journal.pone.0175842 (PMC5433701; doi:10.1371/journal.pone.0175842)
Supplement: S3 Fig — NT8-13 agonist (saturating concentration of 100 nM) and antagonist SR142948 (saturating concentration of 100 nM) were injected subsequently over the NTS1-H4 receptor-coated surface. No binding of SR142948 was detected on the NTS1-H4 receptor that had been saturated previously by agonist NT8-13 peptide, indicating complete occupancy of the binding site and binding to the same binding site. (PDF) [file pone.0175842.s003.pdf]

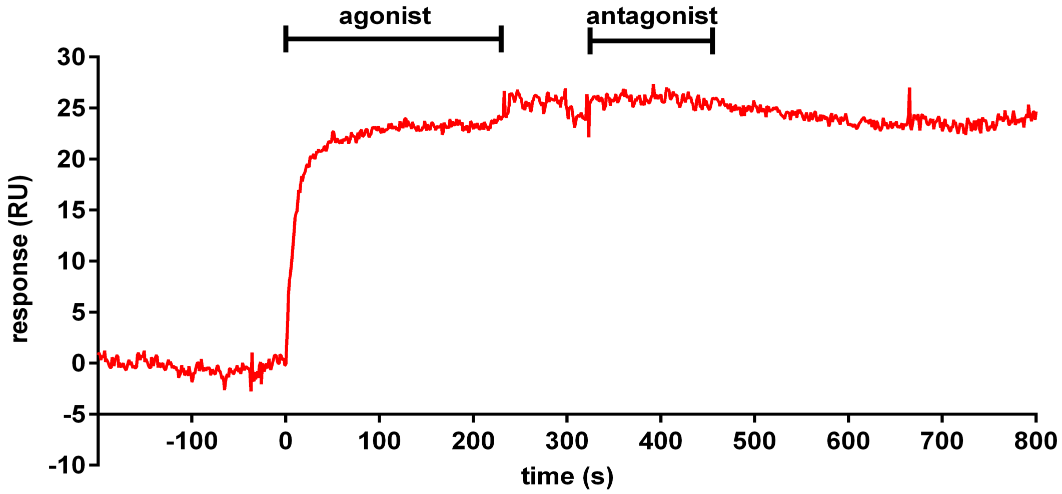

S1 Fig. Competition of neurotensin peptide $NT_{8\text{-}13}$ and antagonist SR142948 on the NTS1-H4 receptor. $NT_{8\text{-}13}$ agonist (saturating concentration of 100 nM) and antagonist SR142948 (saturating concentration of 100 nM) were injected subsequently over the NTS1-H4 receptor-coated surface. No binding of SR142948 was detected on the NTS1-H4 receptor that had been saturated previously by agonist $NT_{8\text{-}13}$ peptide, indicating complete occupancy of the binding site and binding to the same binding site.
