# Supplementary material for: SPR-based fragment screening with neurotensin receptor 1 generates novel small molecule ligands
Source: PLoS One. 2017 May 16;12(5):e0175842. doi: 10.1371/journal.pone.0175842 (PMC5433701; doi:10.1371/journal.pone.0175842)
Supplement: S4 Fig — (A) Overlay of four binding curves monitored for NT8-13A11 on a NTS1-H4 receptor-immobilized surface. (B) Stability plot monitored for NTS1-H4 receptor with NT8-13A11 over 24 hours. Dots in the diagram represent the amplitude of SPR signals observed at the end of the association phase for NT8-13A11 on the binding active (red filled dots) and blocked (red empty dots) NTS1-H4 receptor surface. Empty black diamonds and empty black squares (superimposing signals) represent signals monitored by buffer injections over active and blocked NTS1-H4 receptor surface, respectively. Blocking of the orthosteric binding site in NTS1-H4 receptor on the reference channel was performed by injection of NT8-13 peptide. (PDF) [file pone.0175842.s004.pdf]

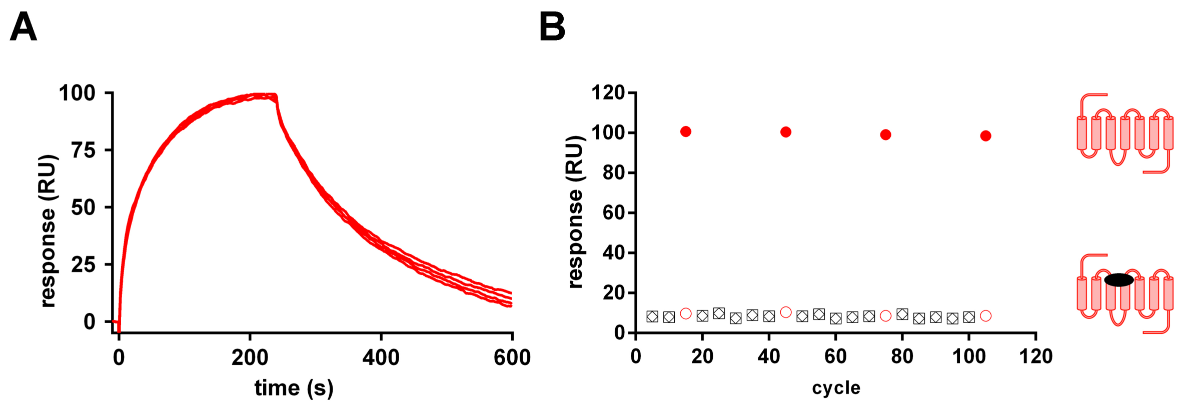

S1 Fig. Stability of captured NTS1-H4 receptor. (A) Overlay of four binding curves monitored for $NT_{8\text{-}13}A_{11}$ on a NTS1-H4 receptor-immobilized surface. (B) Stability plot monitored for NTS1-H4 receptor with $NT_{8\text{-}13}A_{11}$ over 24 hours. Dots in the diagram represent the amplitude of SPR signals observed at the end of the association phase for $NT_{8\text{-}13}A_{11}$ on the binding active (red filled dots) and blocked (red empty dots) NTS1-H4 receptor surface. Empty black diamonds and empty black squares (superimposing signals) represent signals monitored by buffer injections over active and blocked NTS1-H4 receptor surface, respectively. Blocking of the orthosteric binding site in NTS1-H4 receptor on the reference channel was performed by injection of $NT_{8\text{-}13}$ peptide.
