# Supplementary material for: SPR-based fragment screening with neurotensin receptor 1 generates novel small molecule ligands
Source: PLoS One. 2017 May 16;12(5):e0175842. doi: 10.1371/journal.pone.0175842 (PMC5433701; doi:10.1371/journal.pone.0175842)
Supplement: S5 Fig — (PDF) [file pone.0175842.s005.pdf]

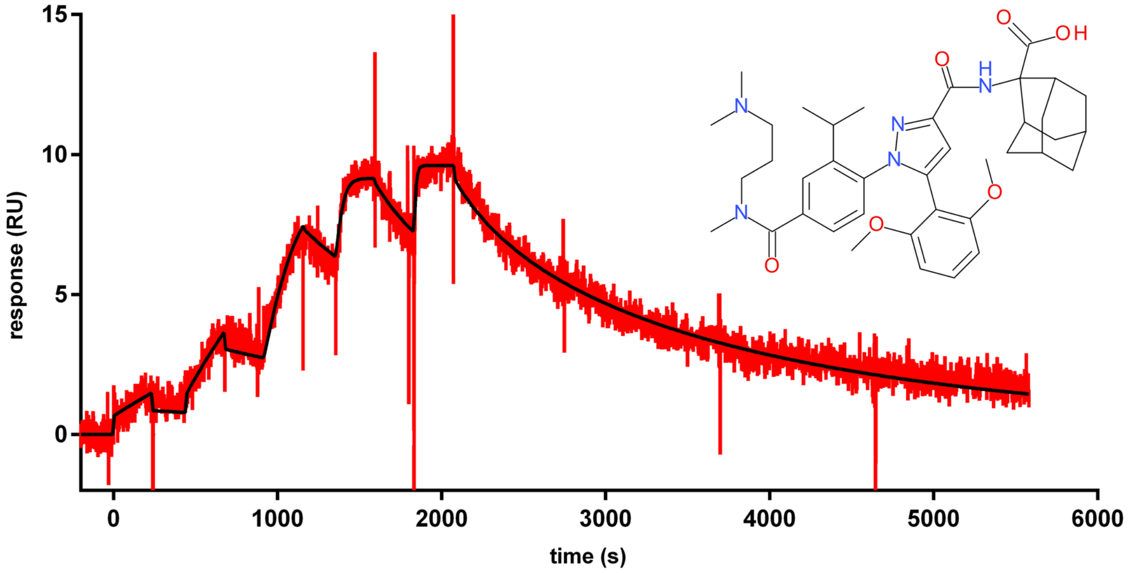


S1 Fig. Titration of SR142948 antagonist up to 25 nM over the NTS1-H4 surface monitored in single cycle kinetic mode (red curve) overlaid with the calculated curve for a one-to-one interaction (black curve) and structure of the SR142948 antagonist.
