# Supplementary material for: SPR-based fragment screening with neurotensin receptor 1 generates novel small molecule ligands
Source: PLoS One. 2017 May 16;12(5):e0175842. doi: 10.1371/journal.pone.0175842 (PMC5433701; doi:10.1371/journal.pone.0175842)
Supplement: S6 Fig — 99.7% of fragments in the library have a molecular mass below 350 Da. (PDF) [file pone.0175842.s006.pdf]

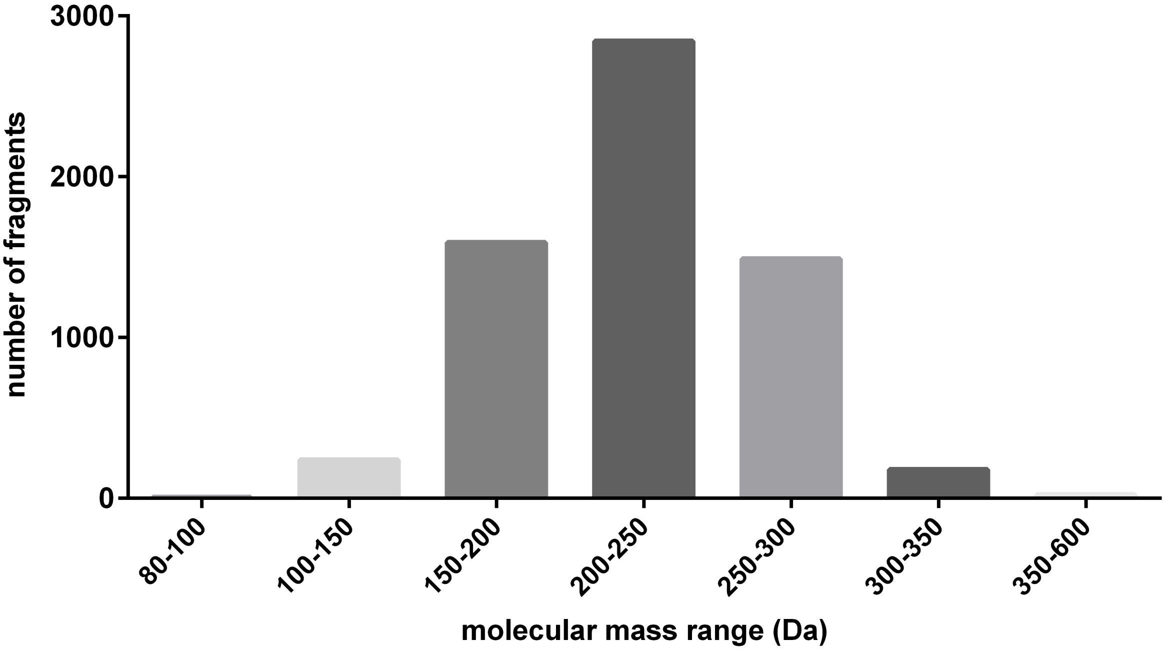

S1 Fig. Distribution of molecular mass of the fragments within the Roche fragment library comprising 6369 structures. 99.7% of fragments in the library have a molecular mass below 350 Da.
