# Supplementary material for: SPR-based fragment screening with neurotensin receptor 1 generates novel small molecule ligands
Source: PLoS One. 2017 May 16;12(5):e0175842. doi: 10.1371/journal.pone.0175842 (PMC5433701; doi:10.1371/journal.pone.0175842)
Supplement: S7 Fig — (A) Typical binding curve for a fragment demonstrating fast kinetics (fast association, saturation of signal in the association phase (signal plateau) and fast dissociation, returning to baseline). (B) Promiscuous fragments: stickiness of compound or atypical sensorgrams (signal lowering in the association phase and signal dropping below baseline in the dissociation phases). (PDF) [file pone.0175842.s007.pdf]

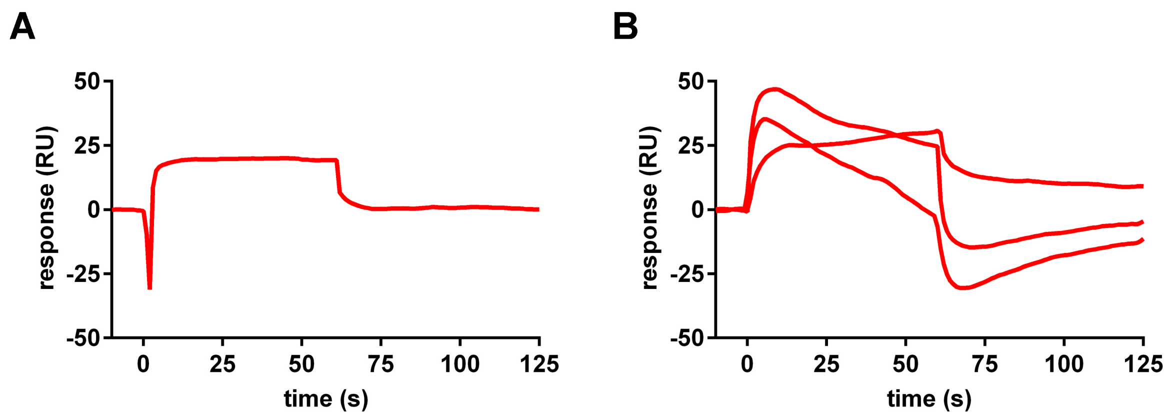


**S1 Fig. Binding curves monitored by the pre-cleaning process of the fragment library.** (A) Typical binding curve for a fragment demonstrating fast kinetics (fast association, saturation of signal in the association phase (signal plateau) and fast dissociation, returning to baseline). (B) promiscuous fragments: stickiness of compound or atypical sensorgrams (signal lowering in the association phase and signal dropping below baseline in the dissociation phases).
