# Supplementary material for: SPR-based fragment screening with neurotensin receptor 1 generates novel small molecule ligands
Source: PLoS One. 2017 May 16;12(5):e0175842. doi: 10.1371/journal.pone.0175842 (PMC5433701; doi:10.1371/journal.pone.0175842)
Supplement: S8 Fig — Among 44 SPR-confirmed hits, 13 clusters and 9 singletons were identified. 8 hits representing 4 clusters and 1 singleton were selected for NMR validation. 4 hits validated by NMR represent 2 clusters and 1 singleton. (PDF) [file pone.0175842.s008.pdf]

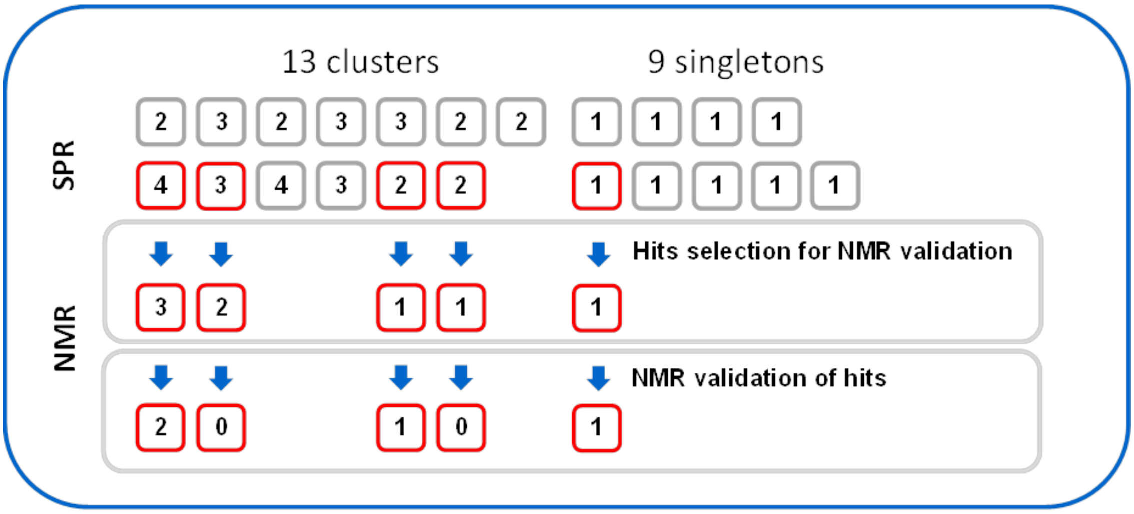

S8 Fig. Clustering of SPR hits and hit selection for validation by NMR. Among 44 SPR-confirmed hits, 13 clusters and 9 singletons were identified. 8 hits representing 4 clusters and 1 singleton were selected for NMR validation. 4 hits validated by NMR represent 2 clusters and 1 singleton.
