# Supplementary material for: SPR-based fragment screening with neurotensin receptor 1 generates novel small molecule ligands
Source: PLoS One. 2017 May 16;12(5):e0175842. doi: 10.1371/journal.pone.0175842 (PMC5433701; doi:10.1371/journal.pone.0175842)
Supplement: S9 Fig — (A) The superposition of the peptidic agonist (X-ray structure [4] shown as ball and stick, with carbon atoms colored in pink) with the antagonist SR142948 (docked conformation shown as ball and stick, with carbon atoms colored in blue) in NTS1-H4 (shown as stick, with carbon atoms colored in green) binding pocket (shown as the molecular surface colored as white). Nitrogen atoms are colored in blue and oxygen atoms are colored in red in both ligand and protein. The same rules are applied for the following figures. (B) The binding mode of fragment hit 1 (docked conformation shown as ball and stick, with carbon atoms colored in orange) in NTS1-H4 binding pocket. Yellow dash line represents hydrophobic interaction, red dash line represents electrostatic interaction, and orange dash line represents π-π interaction. The same rules are applied for the following figure. (C) The binding mode of fragment hit 3 (docked conformation shown as ball and stick, with carbon atoms colored in orange) in NTS1-H4 binding pocket. (PDF) [file pone.0175842.s009.pdf]

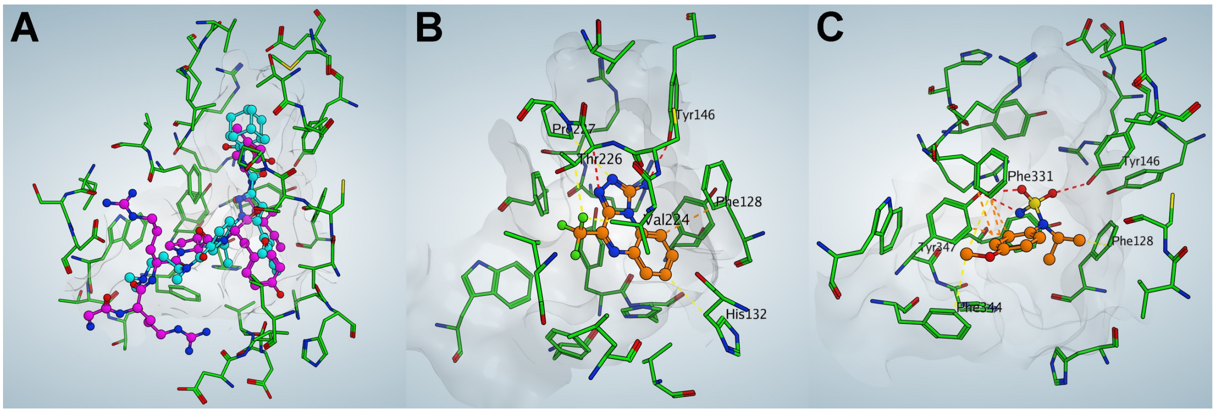

S9 Fig. The superposition of the peptidic agonist (NT)8-13 with the antagonist SR142948 as well as fragment structures as derived from docking experiments. (A) The superposition of the peptidic agonist (X-ray structure [4] shown as ball and stick, with carbon atoms colored in pink) with the antagonist SR142948 (docked conformation shown as ball and stick, with carbon atoms colored in blue) in NTS1-H4 (shown as stick, with carbon atoms colored in green) binding pocket (shown as the molecular surface colored as white). Nitrogen atoms are colored in blue and oxygen atoms are colored in red in both ligand and protein. The same rules are applied for the following figures. (B) The binding mode of fragment hit 1 (docked conformation shown as ball and stick, with carbon atoms colored in orange) in NTS1-H4 binding pocket. Yellow dash line represents hydrophobic interaction, red dash line represents electrostatic interaction, and orange dash line represents [illegible]interaction. The same rules are applied for the following figure. (C) The binding mode of fragment hit 3 (docked conformation shown as ball and stick, with carbon atoms colored in orange) in NTS1-H4 binding pocket.
