# Supplementary material for: SPR-based fragment screening with neurotensin receptor 1 generates novel small molecule ligands
Source: PLoS One. 2017 May 16;12(5):e0175842. doi: 10.1371/journal.pone.0175842 (PMC5433701; doi:10.1371/journal.pone.0175842)
Supplement: S1 File — (DOCX) [file pone.0175842.s010.docx]

**Title long: SPR-based Fragment Screening with biophysically stabilized Neurotensin Receptor 1 generates novel small molecule ligands**

**Authors**

Sylwia Huber^1^, Fabio Casagrande^1^, Melanie N. Hug^1^, Lisha Wang^1^, Philipp Heine^2^, Lutz Kummer^2,3#b^, Andreas Plückthun^2^, Michael Hennig^1,#a4^

**Affiliations**

^1^Roche Innovation Center Basel, Pharmaceutical Research and Early Development, 4070 Basel, Switzerland

^2^Department of Biochemistry, University of Zurich, 8057 Zurich, Switzerland

^3^Present address: G7 Therapeutics AG, 8952 Schlieren, Switzerland

^#a^Current Address: leadXpro AG, PARK INNOVAARE, 5234 Villigen, Switzerland

^#b^Current Address: G7 Therapeutics AG, 8952 Schlieren, Switzerland

**Supporting Information**

**Cloning of GPCR expression construct**

The rNTS_1_ variant NTS_1_-H4^34^ was expressed in *E. coli* under a *lac* promoter using the a derivative of the vector pRG/III-hsMBP^46^ (kindly provided by R. Grisshammer (National Institute of Neurological Disorders and Stroke, National Institutes of Health, Rockville, MD)). NTS_1_-H4 was N-terminally truncated at amino acid E44 (sequential NTS_1_ numbering) and linked via a human rhinovirus (HRV) 3C protease site to a hexa-histidine tag and maltose-binding protein (MBP). At the C-terminus the receptor was truncated at amino acid G390 and fused via a hexa-glycine-serine linker to an avi-tag, followed by another HRV 3C protease site, a penta-asparagine and a di-glycine-serine linker to thioredoxin A (TrxA) and a deca-histidine tag. Amino acids E273-T290 of the intracellular loop 3 were deleted and the two potential free cysteines C386 and C388 at the C-terminus of the receptor were both mutated to alanine. Truncations and removal of cysteine residues were carried out to facilitate protein purification, protein crystallization and biophysical experiments without comprising signaling functionality of NTS_1_-H4^4^.

**Protein expression**

*Escherichia coli* BL21 cells were transformed with the expression plasmid harboring the NTS_1_ variant NTS_1_-H4^4^ and grown overnight at 37°C in 1 L 2YT medium supplemented with 1% (w/v) glucose and 100 µg/mL ampicillin. A fermenter (Bioengineering D 558) containing 50 L 2YT, 0.5% (w/v) glucose, and 100 µg/ml ampicillin was inoculated using the entire pre-culture and grown to an OD_600_ of 2.5 at 37°C. Receptor expression was induced with 1 mM isopropyl-β-D-thiogalactopyranoside (IPTG) and cells were cultivated at 28°C overnight. In addition, 1 µM biotin was added after induction to ensure efficient *in vivo* biotinylation of expressed NTS_1_-H4 harboring a C-terminal avi-tag. Subsequent to overnight expression, cells were harvested and cell pellets were frozen in liquid nitrogen and stored at -80°C.

**Protein purification**

For one batch purification, 25 g of frozen *E. coli* pellet were used. Cells were thawed at room temperature and resuspended in 100 ml in solubilization buffer, containing 100 mM HEPES pH 8.0, 20% (v/v) glycerol and 400 mM NaCl. All following steps were carried out at 4°C. 0.5 mL of 1 M MgCl_2_ (5 mM), 2 mg DNase I, 200 mg lysozyme, and 20 mL of a detergent mixture composed of 0.2% (w/v) cholesteryl hemisuccinate Tris salt (CHS) and 2% (w/v) dodecyl-ß-D-maltopyranoside (DDM) were added to the cells. The mixture was incubated for 30 min, followed by cell lysis via mild sonification for 30 min in an ice-water bath. After cell lysis, 0.4 ml of 5 M imidazole was added and the mixture was incubated for another 30 min. The suspension was centrifuged for 30 min at 28000 *g*. The supernatant was mixed with 5 mL of TALON resin (Clontech, Mountain View, CA, USA), which had been pre-equilibrated with IMAC binding buffer (25 mM HEPES pH 8.0, 10% (v/v) glycerol, 600 mM NaCl, 0.3% (w/v) DDM and 15 mM imidazole) and incubated for 2 h on a rolling device. Subsequently, the mixture was loaded into an empty PD10 column (GE Healthcare, Uppsala, Sweden) and was washed with 50 mL of IMAC binding buffer. Elution of bound protein was performed with 15 ml IMAC elution buffer containing 25 mM Hepes pH 8.0, 10% (v/v) glycerol, 150 mM NaCl, 0.3% (w/v) DDM and 250 mM imidazole. 500 µL of 1.6 mg/mL HRV 3C protease was added to the elution and incubated for 1 h at 4°C, followed by addition of 250 µL 10% (w/v) L-MNG and incubation for 1 h at 4°C. The cleaved protein was diluted threefold with SP binding buffer containing 10 mM HEPES pH 7.0, 10% (v/v) glycerol, and 0.01% (w/v) L-MNG, and was loaded into a PD10 column filled 2.5 ml SP Sepharose beads pre-equilibrated with SP binding buffer. The resin was washed with 15 ml SP binding buffer, followed by 12.5 ml SP wash buffer (10 mM HEPES pH 7.7, 10% (v/v) glycerol, 35 mM NaCl, and 0.01% (w/v) L-MNG, and another 2 mL SP binding buffer. NTS_1_-H4 was eluted with 12 mL SP elution buffer containing 10 mM HEPES pH 7, 10% (v/v) glycerol, 350 mM NaCl, and 0.01% (w/v) L-MNG. Eluted receptor was concentrated in an Amicon-15 Ultra concentrator with a 50 kDa cutoff to a final volume of less than 1 mL. Lastly, the NTS_1_-H4 was subjected to preparative size exclusion chromatography using a Superdex 200 10/300 GL column (GE Healthcare, Uppsala, Sweden), which had been pre-equilibrated with 10 mM HEPES pH 8, 150 mM NaCl, and 0.01% (w/v) L-MNG. Peak fractions corresponding to NTS_1_-H4 were pooled (final volume 3-4 mL) and concentrated in an Amicon-4 Ultra concentrator (Millipore, Billerica, MA, USA) with a 50 kDa cutoff to a final protein concentration of approximately 50 µM. Purified and concentrated NTS_1_-H4 was mixed with a glycerol buffer (10 mM HEPES pH 8, 150 mM NaCl, 0.01% (w/v) L-MNG, and 50% (v/v) glycerol) to yield a final glycerol concentration of 25%, aliquoted into 20 µL samples and frozen in liquid nitrogen. Receptor aliquots were stored at -80°C for later usage.

Supporting Information

S1 Fig. Capturing of NTS1-H4, biotinylated at the C-terminal avi-tag, on the biosensor overlay of 8 sensorgrams monitored on a Biacore A4000® during the capturing of the NTS1-H4 receptor on streptavidin pre-coated SA sensor. The resonance signal was monitored on eight spots in four flow channels (two spots per flow channel) in parallel. NTS1-H4 receptor was contacted twice (twice 20 min at a receptor concentration of 1 µM) with the sensor surface to achieve protein densities of ~9000 RUs. Finally, a biotin solution at 500 µM was injected over the sensor surface to block remaining free binding sites on streptavidin.

S2 Fig. Binding of NT_8-13_A_11,12_ to NTS1-H4. (A) dose-response titration of NT_8-13_A_11,12_ over high density NTS1-H4 surface monitored by peptide titration up to 500 nM (dilution factor 2). (B) Sigmoidal dose-response curve and mathematical fit for one-to-one interaction with a maximal signal calculated theoretically. The apparent affinity constant (K_D_) was estimated to be 90 nM.

S3 Fig. Competition of neurotensin peptide NT_8-13_ and antagonist SR142948 on the NTS1-H4 receptor. NT_8-13_ agonist (saturating concentration of 100 nM) and antagonist SR142948 (saturating concentration of 100 nM) were injected subsequently over the NTS1-H4 receptor-coated surface. No binding of SR142948 was detected on the NTS1-H4 receptor that had been saturated previously by agonist NT_8-13_ peptide, indicating complete occupancy of the binding site and binding to the same binding site.

S4 Fig. Stability of captured NTS1-H4 receptor. (A) Overlay of four binding curves monitored for NT_8-13_A_11_ on a NTS1-H4 receptor-immobilized surface. (B) Stability plot monitored for NTS1-H4 receptor with NT_8-13_A_11_ over 24 hours. Dots in the diagram represent the amplitude of SPR signals observed at the end of the association phase for NT_8-13_A_11_ on the binding active (red filled dots) and blocked (red empty dots) NTS1-H4 receptor surface. Empty black diamonds and empty black squares (superimposing signals) represent signals monitored by buffer injections over active and blocked NTS1-H4 receptor surface, respectively. Blocking of the orthosteric binding site in NTS1-H4 receptor on the reference channel was performed by injection of NT_8-13_ peptide.

S5 Fig. Titration of SR142948 antagonist up to 25 nM over the NTS1-H4 surface monitored in single cycle kinetic mode (red curve) overlaid with the calculated curve for a one-to-one interaction (black curve) and structure of the SR142948 antagonist.

S6 Fig. Distribution of molecular mass of the fragments within the Roche fragment library comprising 6369 structures. 99.7% of fragments in the library have a molecular mass below 350 Da.

S7 Fig.  Binding curves monitored by the pre-cleaning process of the fragment library. (A) Typical binding curve for a fragment demonstrating fast kinetics (fast association, saturation of signal in the association phase (signal plateau) and fast dissociation, returning to baseline). (B) Promiscuous fragments: stickiness of compound or atypical sensorgrams (signal lowering in the association phase and signal dropping below baseline in the dissociation phases).

S8 Fig. Clustering of SPR hits and hit selection for validation by NMR. Among 44 SPR-confirmed hits, 13 clusters and 9 singletons were identified. 8 hits representing 4 clusters and 1 singleton were selected for NMR validation. 4 hits validated by NMR represent 2 clusters and 1 singleton.

S9 Fig. The superposition of the peptidic agonist (NT)_8-13_ with the antagonist SR142948 as well as fragment structures as derived from docking experiments. (A) The superposition of the peptidic agonist (X-ray structure [4] shown as ball and stick, with carbon atoms colored in pink) with the antagonist SR142948 (docked conformation shown as ball and stick, with carbon atoms colored in blue) in NTS1-H4 (shown as stick, with carbon atoms colored in green) binding pocket (shown as the molecular surface colored as white). Nitrogen atoms are colored in blue and oxygen atoms are colored in red in both ligand and protein. The same rules are applied for the following figures. (B) The binding mode of fragment hit 1 (docked conformation shown as ball and stick, with carbon atoms colored in orange) in NTS1-H4 binding pocket. Yellow dash line represents hydrophobic interaction, red dash line represents electrostatic interaction, and orange dash line represents π-π interaction. The same rules are applied for the following figure. (C) The binding mode of fragment hit 3 (docked conformation shown as ball and stick, with carbon atoms colored in orange) in NTS1-H4 binding pocket.
